# Temperature dependence of the electrical transport properties in few-layer graphene interconnects

**DOI:** 10.1186/1556-276X-8-335

**Published:** 2013-07-25

**Authors:** Yanping Liu, Zongwen Liu, Wen Siang Lew, Qi Jie Wang

**Affiliations:** 1 NOVITAS, Nanoelectronics Centre of Excellence, School of Electrical & Electronic Engineering, Nanyang Technological University, 50 Nanyang Ave, Singapore 639798, Singapore; 2 School of Chemical and Biomolecular Engineering, The University of Sydney, New South Wales 2006, Australia; 3 School of Physical and Mathematical Sciences, Nanyang Technological University, 21 Nanyang Link, Singapore 637371, Singapore; 4 Centre for Disruptive Photonic Technologies, School of Physical and Mathematical Sciences, Nanyang Technological University, 21 Nanyang Link, Singapore 637371, Singapore

**Keywords:** Graphene, Graphene multilayer, Short-range scattering theory, Coulomb scattering effect

## Abstract

We report a systematic investigation of the temperature dependence of electrical resistance behaviours in tri- and four-layer graphene interconnects. Nonlinear current–voltage characteristics were observed at different temperatures, which are attributed to the heating effect. With the resistance curve derivative analysis method, our experimental results suggest that Coulomb interactions play an essential role in our devices. The room temperature measurements further indicate that the graphene layers exhibit the characteristics of semiconductors mainly due to the Coulomb scattering effects. By combining the Coulomb and short-range scattering theory, we derive an analytical model to explain the temperature dependence of the resistance, which agrees well with the experimental results.

## Background

Graphene has been a subject of intense research since it was discovered in 2004 because of its intriguing band Structure [1,2]. The energy-momentum relationship of graphene is found to be linear for low energies near the six corners of the two-dimensional (2D) hexagonal Brillouin zone [1-4], leading to a zero effective mass for electrons and holes [2-5]. Such behaviour can be described by the Dirac equation for spin 1/2 particles [1-6]. Furthermore, graphene is also an excellent electronic material as it can be either a metal or semiconductor depending on the edge states, zigzag or armchair [7]. It exhibits superior mobility, with reported values in excess of 15,000 cm^2^ V^−1^ s^−1^[1], which is superior to that of III-V semiconductors for high-speed device applications. As such, graphene has been widely predicted to be a potential material for post-complimentary metal-oxide semiconductor technology, particularly for use as ballistic transistors or interconnects [8-12]. Most graphene studies have focused on monolayer structures [1]. Recently, few-layer graphene (FLG) have received much attention because of its promising bandgap tunability. For instance, bilayer graphene is reported to have a tunable bandgap [13,14] and trilayer graphene is a semimetal in the ideal case with a gate-tunable overlapped bandgap [15]. As more graphene layers are added, the electrical properties of FLG also change, which can be further explored for the design of various devices [15]. However, theoretical understanding and experimental investigations of FLG are still lacking for applications such as interconnect.

In this letter, we report a systematic investigation of the temperature dependence behaviour of the four-terminal electrical resistance in FLG interconnects. The resistance of tri- and four-layer graphene, under direct current (DC) electric fields and in a temperature range from 5 to 340 K was measured. The T^-1/2^ dependence shows the evidence of the electron–electron Coulomb interaction in FLG. Our temperature-dependent resistance results reveal that the FLG interconnects display semiconductor properties and further confirm that Coulomb interaction can play a dominant role.

## Methods

**Figure 1:**
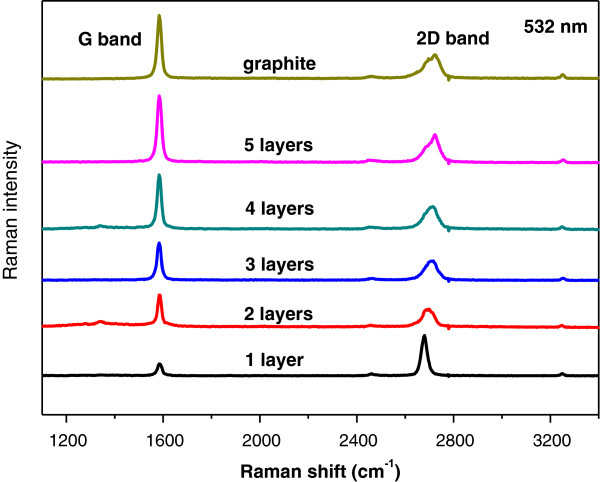
**Comparison of Raman spectra at 532 nm for few-layer graphene.** The position of G peak and the spectral features of 2D band confirm the number of atomic layer of the graphene devices.

The graphene layers were produced by mechanical exfoliation techniques [2] from bulk highly oriented pyrolitic graphite and then transferred onto a Si/SiO_2_ substrate. The number of graphene layers was confirmed by micro-Raman spectroscopy through the 2D-band deconvolution procedure [16]. The Raman spectra of the graphene structures were measured at room temperature using a WITec CRM200 instrument (Ulm, Germany) under a 532-nm excitation wavelength in the backscattering configuration [16,17]. Shown in Figure 1 is the Raman spectrum with clearly distinguishable G band and 2D band. The number of graphene layers is distinguished from the full-width half maximum of the 2D band peak [17]. Optical photolithography technique was used to pattern four terminal Cr/Au contact pads on the graphene structures. The metallic contact electrodes of Cr and Au films of thickness 5 and 80 nm, respectively, were deposited using thermal evaporation techniques at a vacuum pressure of 2 × 10^−7^ mbar. Electronic transport measurements were performed on multiple samples, using the physical property measurement system (PPMS, Quantum Design, San Diego, CA, USA) with a fixed excitation current of 0.01 mA; the temperature varied from 5 to 340 K.

## Results and discussion

**Figure 2:**
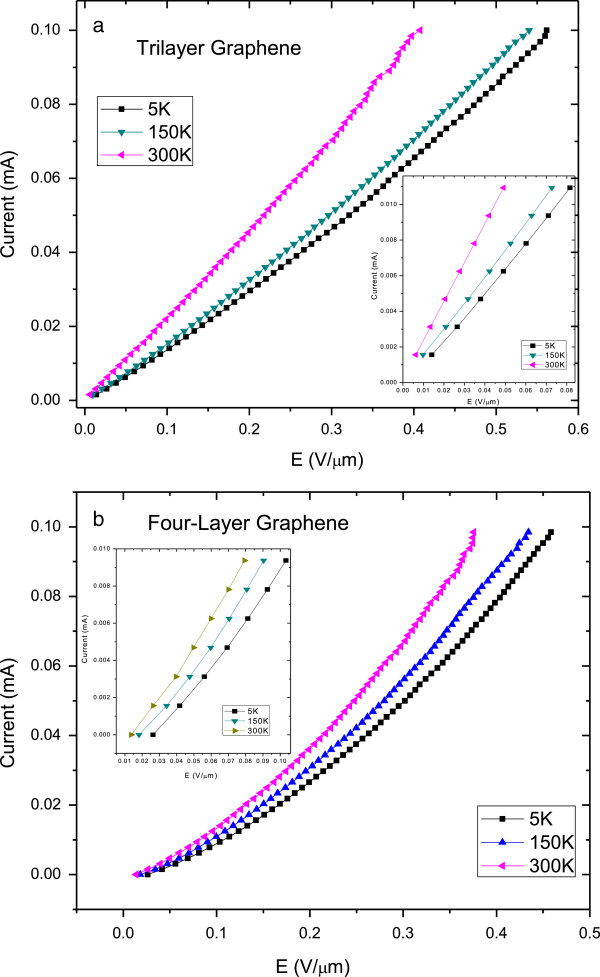
**Temperature-dependent current–voltage characteristics of (a) tri- and (b) four-layer graphene interconnects.** Insets show the details of the low electric field measurements. For tri- and four-layer graphene, resistors show sublinear characteristics at low field (<0.01 V/μm) and superlinear *I-V* curve for the high field due to the heat effect.

Figure 2 shows the representative current–voltage (*I-V)* characteristics at different temperatures of (a) tri- and (b) four-layer graphene interconnects. Insets show the enlargement of the measurement results at low electric fields. For the tri- and four-layer graphene, the interconnect resistors display two distinct regions of ohmic characteristic: one at fields larger than 0.01 V/μm but less than 0.10 V/μm and the other at fields larger than 0.10 V/μm. The nonlinear behaviour of current–voltage characteristics at low threshold (<0.10 V/μm), and the second ohmic region in the strong DC electric field (>0.10 V/μm) can be explained by the heating effect [18]. Within a strong DC electric field, the relaxation grows sharply with heating, and the recombination of carriers is dominant as compared to thermal generation [18,19]. At sufficiently high DC electric field, we observe linear *I-V* over the whole temperature measurement range.

**Figure 3:**
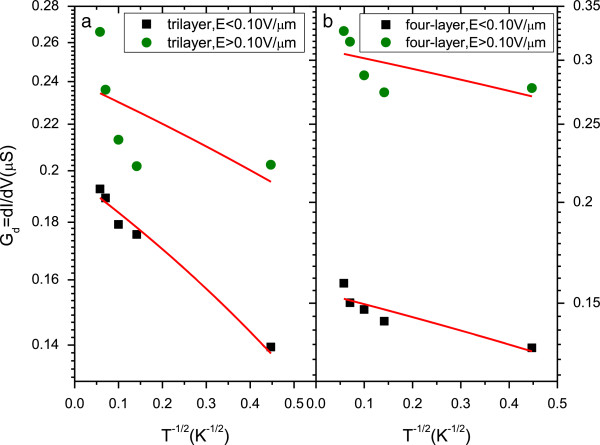
**Differential conductance**$G_{d}=\frac{\mathrm{dI}}{\mathrm{dV}}$**of (a) tri- and (b) four-layer graphene as function of temperature *T***^**−1/2**^**.** The fit line shows good consistency with the Efros-Shklovskii (ES) variable-range-hopping (VRH) model at the low DC electric field, and the results clearly indicate that Coulomb scattering is the dominant scattering mechanism in our system.

In order to study the existence of electron–electron Coulomb interaction and how it plays an important role in our system, we adopted the resistance curve derivative analysis (RCDA) method to investigate the dominant scattering mechanism [20]. Figure 3 shows the differential conductance *G*_*d*_ = *dI*/*dV* of (a) tri- and (b) four-layer graphene as function of the temperature *T*^−1/2^ on a semi-logarithmic scale. As shown in the Figure 3, we can see the experiment results can be well fitted with the Efros-Shklovskii (ES) variable-range-hopping (VRH) model at the low DC electric field. One should note that, for the high electric field conductance, the fitted line shows some deviation from the model due to the heating effect. Therefore, our data suggest that Coulomb scattering is the main scattering mechanism in our device.

**Figure 4:**
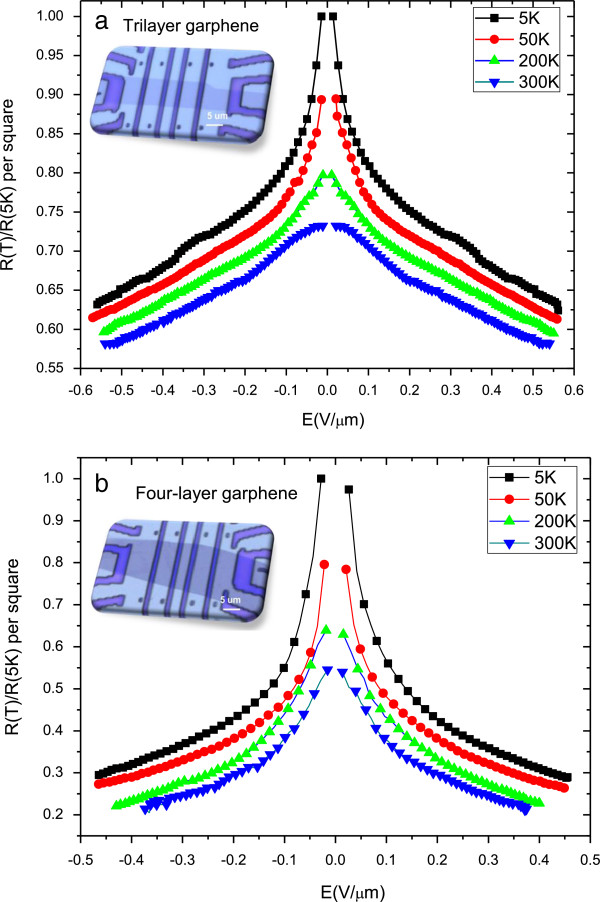
**Dimensionless resistance, *R*(*T*)/*R*(5*K*), versus electric field *E *at different temperatures for (a) tri- and (b) four-layer graphene.** The resistance of graphene interconnects drops substantially as the electric field is increasing; the corresponding resistance drop is larger for low temperatures. Inset is an optical micrograph of the tri- and four-layer graphene with four Cr/Au contact electrodes, respectively.

There are many experimental and theoretical reports on the mobility of one- to three-layer graphene from two- and four-terminal measurements. The main scattering mechanisms in one- to three-layer graphene are Coulomb scattering [21-23], short-range scattering [24] and phonon scattering by graphene phonon [25]. To further study the scattering mechanism in our device, we investigated temperature-dependent resistance as a function of the electric field *E*. Shown in Figure 4 are the dimensionless resistance *R*_*T*_/*R*_*T* = 5*K*_ as a function of the electric field *E* at different temperatures, for (a) tri- and (b) four-layer graphene interconnects. Insets display the optical micrographs of the FLG interconnect. At a lower temperature range of 5 to 50 K, as the electric field increases from 0 to 0.6 V/μm, the resistance of the tri- and four-layer graphene interconnects show a reduction of about 30% and 70%, respectively. However, for the temperature range *T* ≥ 200 K, the corresponding resistance drop is smaller. The larger drops of the resistance at lower temperature range indicate that Coulomb scattering is the main transport mechanism in the FLG interconnects at this temperature range as it is proportional to the carrier density. Hence, Coulomb scattering is strongly dependent on temperature. We further note that with increasing temperature, the observed results indicate that the scattering induced by electric field from the substrate surface polar phonons is significantly screened by the additional graphene layers at room temperature [21,22].

**Figure 5:**
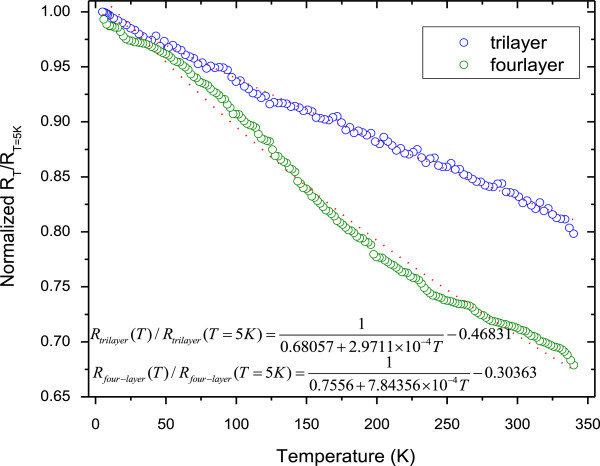
**Normalized electrical resistance per square measurements as function of temperature of tri- and four-layer graphene interconnects.** The results show that when the temperature increases from 5 to 340 K, the resistance of the tri- and four-layer graphene interconnects drops significantly, indicating a semiconductor property of the graphene. The symbols are the measured data, and the lines are fits.

In order to further study the VRH and localized insulating behaviour, we investigate temperature dependence of electronic transport measurements on a tri- and four-layer graphene. Figure 5 shows the temperature dependence of the resistance measurement of the tri- and four-layer graphene. We define the relative change in resistance normalized by the temperature at 5 K: *R*_*T*_/*R*_*T* = 5*K*_, whereby we investigate the temperature dependence change of the resistance. In Figure 5, we present the electrical resistance of the three and four layers of graphene interconnects as a function of temperature. The results show that an appreciable monotonic increase of *R*_*T*_/*R*_*T* = 5*K*_ is observed for decreasing temperature for both the tri- and four-layer graphene. This *R-T* behaviour indicates that the carriers transport in the graphene layers is non-metallic in nature. This implies that, the resistance does not originate from thermal activation but is attributed to ES VRH between localized states induced by the charge impurities [20-23]. This is the further evidence that the electron–electron Coulomb interaction effects are prominent in such system. As the temperature is reduced from 340 to 5 K, the increase of the four-layer graphene resistance is much larger, which is around 40%, compared to the trilayer graphene, which is found to be 20%.

At low temperature, the main scattering mechanisms in graphene are largely due to the Coulomb impurity and the short-range defect scatterings [24]. Based on Matthiessen’s rule, the overall mobility can be written as [22]:

(1) $$\left(\frac{1}{\mu_{\mathrm{total}}}=\frac{1}{\mu_{C}}+\frac{1}{\mu_{\mathrm{sr}}}\right)$$

Based on a model proposed by Hwang et al. [24], we can assume that the scattering centres of charge are at the SiO_2_-graphene interface, and the short-range scattering is constant. Then the energy average scattering time is deduced as [21,22]:

(2) $$\langle \tau \rangle =\frac{\int dE_{k}E_{k}\tau \left(E_{k}\right)\left(-\frac{\partial f}{\partial E_{K}}\right)}{\int dE_{k}E_{k}\left(-\frac{\partial f}{\partial E_{K}}\right)},$$

where *E*_*k*_ is the wave vector energy and *τ*(*E*_*k*_) is the transport scattering rate. For the low temperature limit, the scattering time averaged over energy can be written as 〈*τ*〉 ≈ *τ*(*E*_*F*_) [21]. The density of states *D*(*E*_*F*_) in tri- and four-layer graphene is assumed to be a constant $D\left(E_{F}\right)=\frac{2m^{*}}{\pi \hbar^{2}}$. Here, the Fermi energy is $E_{F}=\frac{\hbar^{2}k^{2}}{2m^{*}}=\frac{\hbar^{2}\mathrm{\pi n}}{2m^{*}}$, and based on the Boltzmann equation of mobility as function of the scattering time: $\mu =\frac{\mathrm{eD}\left(E_{F}\right)v_{F}^{2}\left(\tau \right)}{2n}$, we can obtain the mobility of graphene as $\mu_{3-4\mathrm{layers}}=\frac{e\langle \tau \rangle }{m^{*}}$. As such, at low temperatures, the Coulomb scattering is proportional to the carrier density in the tri- and four-layer graphene structures [21-23].

In the high temperature regime, the Coulomb scattering is a strong function of temperature while the short-range scattering is independent of temperature. This is attributed to the density of states, the matrix element of graphene and the screening function being energy independent in FLG [21-23]. Hence, the mobility increases proportionally with the temperature (μ_3-4 layers_ ∝ *k*_*B*_*T*) [21]. For tri- and four-layer graphene, the resistance can be expressed as:

(3) $$R_{3-4\mathrm{layers}}=R_{C}{}_{-3–4\mathrm{layers}}+R_{\mathrm{sr}}{}_{-3–4\mathrm{layers}},$$

where we have defined $R_{C-3‒4\;\mathrm{layers}}=\frac{1}{\left(A+\mathrm{BT}\right)\cdot n^{2}e}$, *R*_*sr*−3–4 layers_ = *C*, and A, B and C are the fitting parameters. In our measurements, we have observed a linear approximation for the temperature-dependent normalized resistance of tri- and four-layer graphene:

(4) $$\begin{array}{l} R_{3\;\mathrm{layer}}\left(T\right)/R_{3\;\mathrm{layer}}\left(T=5K\right)\\ \;=\frac{1}{0.68057+2.9711\times 10^{-4}T}-0.46831\; \end{array}$$

(5) $$\begin{array}{l} R_{4\;\mathrm{layer}}\left(T\right)/R_{4\;\mathrm{layer}}\left(T=5K\right)\\ \;=\frac{1}{0.7556+7.84356\times 10^{-4}T}-0.30363\; \end{array}$$

These considerations explain qualitatively why the resistance of tri- and four-layer graphene decreases with the increasing temperature. We note that due to the complexity of the FLG band structure, these anomalous electrical properties are believed to originate in the unusual band structures near the Fermi level of graphene [26-29]. More rigorous theoretical explanation of FLG intrinsic semiconductor behaviours would be interesting and requires further experimental investigations.

## Conclusions

In conclusion, FLG interconnects were fabricated via a combination of mechanical exfoliation and photo lithography techniques. The temperature dependence of the electrical resistance of tri- and four-layer graphene was investigated. The observed *I-V* curve shows unique combination of the low threshold of linearity and manifestation of the second ohmic region for the strong DC electric field in the FLG interconnects. With the RCDA method, our experimental results suggest that Coulomb interaction plays an essential role. The non-metallic temperature-dependent resistance is observed in the temperature range of 5 to 340 K. In this case, even though the FLG band structure as semimetal with zero-band gap, tri- and four-layer graphene resistors behave more like semiconductors. By combining the Coulomb and short-range scattering theories, an analytical model was developed, which well explains the experimental results.

## Competing interests

The authors declare that they have no competing interests.

## Authors’ contributions

YPL fabricated the device and performed the experiments. WQJ and WSL coordinated the project. ZWL and WSL provided key interpretation of the data. QJW and WSL drafted the paper. All authors read and approved the final manuscript.
